# Phenolic Compounds Isolated from *Calea uniflora* Less. Promote Anti-Inflammatory and Antioxidant Effects in Mice Neutrophils (*Ex Vivo*) and in Mice Pleurisy Model (*In Vivo*)

**DOI:** 10.1155/2019/1468502

**Published:** 2019-11-12

**Authors:** Julia Salvan da Rosa, Marcus Vinicius Pereira Dos Santos Nascimento, Eduardo Benedetti Parisotto, Tamires Cardoso Lima, José Roberto Santin, Maique Weber Biavatti, Ariane Zamoner, Eduardo Monguilhott Dalmarco, Tânia Silvia Fröde

**Affiliations:** ^1^Graduate Course of Pharmacy, Center of Health Sciences, Federal University of Santa Catarina (UFSC), Florianópolis, SC, Brazil; ^2^Department of Biochemistry, Center of Biological Sciences, Federal University of Santa Catarina (UFSC), Florianópolis, SC, Brazil; ^3^Department of Pharmaceutical Sciences, Center of Health Sciences, Federal University of Santa Catarina (UFSC), Florianópolis, SC, Brazil; ^4^Núcleo de Investigações Químico-Farmacêuticas, University of Vale do Itajaí (UNIVALI), Itajaí, SC, Brazil

## Abstract

The literature shows that phenolic compounds possess important antioxidant and anti-inflammatory activities; however, the mechanism underlying these effects is not elucidated yet. The genus *Calea* is used in folk medicine to treat rheumatism, respiratory diseases, and digestive problems. In this context, some phenolic compounds were isolated with high purity from *Calea uniflora* Less. and identified as noreugenin (NRG) and *α*-hydroxy-butein (AH-BU). The aim of this study was to analyze the effect of these compounds on cell viability, the activity of myeloperoxidase (MPO), and apoptosis of mouse neutrophils using *ex vivo* tests. Furthermore, the effect of these compounds on the cytokines, interleukin 1 beta (IL-1*β*), interleukin 17A (IL-17A), and interleukin 10 (IL-10), and oxidative stress was investigated by analyzing lipid peroxidation (the concentration of thiobarbituric acid reactive substances (TBARS)) and activities of antioxidant enzymes, superoxide dismutase (SOD), catalase (CAT), and glutathione S-transferase (GST), using a murine model of neutrophilic inflammation. The NRG and AH-BU reduce MPO activity and increase neutrophil apoptosis (*p* < 0.05). These compounds reduced the generation of oxygen reactive species and IL-1*β* and IL-17A levels but increased IL-10 levels (*p* < 0.05). This study demonstrated that NRG and AH-BU show a significant anti-inflammatory effect by inhibiting the MPO activity and increasing neutrophil apoptosis in primary cultures of mouse neutrophils. These effects were at least partially associated with blocking reactive species generation, inhibiting IL-1*β* and IL-17A, and increasing IL-10 levels.

## 1. Introduction

Inflammation is a physiological response triggered by tissue injury or antigenic stimuli. When these stimuli are chronic, they can cause organ damages, which promote the loss of organ functions [1]. This process involves a cascade of events, which includes the release of mediators and the activation of cells that are involved in the repair of damaged tissue [2, 3].

The relationship between oxidative stress and inflammation is well documented. Activated inflammatory cells produce a number of reactive species at the site of inflammation leading to an exaggerated oxidative stress that can initiate intracellular signaling cascade which enhances proinflammatory gene expression [4]. Almost in the same time, it enhances the ROS-detoxifying enzymes that are crucial for cellular redox balance regulation in the physiological and inflammatory processes. Among them, SOD, CAT, and GST are the most important enzymatic agents involved and are responsible to dismutate superoxide radical, break down hydrogen peroxides, and catalyze conjugation of reduced glutathione, respectively [5]. Regardless, these evidences indicate that oxidative stress plays a pathogenic role in chronic inflammatory diseases by increasing the levels of proinflammatory mediators and cytokines [6].

Among the essential cells of the innate immune response to inflammation, neutrophils are the most abundant leukocytes circulating in humans and are the first white cells recruited by the inflammatory local response [7]. During inflammation, neutrophils become activated and their longevity is prolonged, which ensures their presence at the site of inflammatory response. A series of mediators, such as cytokines, enzymes, and effector molecules, play a crucial role in the host defense against inflammation [8, 9].

Although several effective anti-inflammatory drugs that control signs of inflammation have been approved by different pharmaceutical regulatory agencies, the number of adverse effects shows the limitation of their use [10, 11]. Therefore, the search for new treatments that act in the control of the inflammatory process is necessary to develop an efficient therapeutic strategy [12, 13].

*Calea uniflora* Less. is a perennial and subshrub plant found in southern Brazil [14]. This plant, commonly known as arnica or erva-de-lagarto [15, 16], is used in Brazilian folk medicine to treat rheumatism, respiratory diseases, and digestive problems [17, 18]. Some phenolic compounds identified as noreugenin (NRG), a mixture of orobol+butein (OR+BU), and *α*-hydroxy-butein (AH-BU) were isolated from *Calea uniflora* Less. by phytochemical analysis [16]. These phenolic compounds have known antioxidant and anti-inflammatory activities. The mechanisms of these effects proposed by Zhang and Tsao [19] suggest their ability to scavenge free radicals, restore antioxidant enzyme activities, and regulate cytokine-induced inflammation [19]. In this context, there are not many studies regarding the anti-inflammatory and antioxidant activities of these phenolic compounds (NRG and AH-BU) in the literature. In addition, the data previously published by our research group demonstrated that NRG and AH-BU showed a potent anti-inflammatory activity due to the inhibition of inflammatory cells, mainly neutrophils, in a mouse model of pleurisy induced by carrageenan [20].

In this context, the aim of this study was to extend the research on the anti-inflammatory effect of noreugenin and *α*-hydroxy-butein, looking to the security profile of them in experimental conditions. Here, we investigated the effect of these compounds obtained from *Calea uniflora* Less. on cell viability, myeloperoxidase (MPO), apoptosis, and necrosis of murine neutrophils by using *ex vivo* tests as well as on other proinflammatory mediators such as cytokines, interleukin 1 beta (IL-1*β*) and interleukin 17A (IL-17A), and anti-inflammatory such as interleukin 10 (IL-10) by using *in vivo* assays. We also analyzed the action of NRG and AH-BU on oxidative stress by the analysis of lipid peroxidation and activity of antioxidant enzymes (SOD, CAT, and GST) by using *in vivo* assays.

## 2. Materials and Methods

### 2.1. Plant Material

The leaves of *Calea uniflora* Less. were collected in October 2012, in Imbituba, Santa Catarina, Brazil. The identity of the plant material was confirmed by Dr. John F. Pruski (New York Botanical Garden), and a voucher specimen was documented at the Missouri Botanical Garden Herbarium, St. Louis, Missouri, USA, under the number MO-2383317.

### 2.2. Extraction, Isolation, and Identification of the Compounds

The methods used for the collection and extraction of the plant material as well as for the isolation and identification of the chemical constituents were performed as previously described by Lima et al. [16].

In the first study performed by Rosa et al. [20], it was demonstrated that the dichloromethane (DCM) and ethyl acetate (EtOAc) fractions isolated from fresh leaves from *Calea uniflora* Less. exhibited stronger anti-inflammatory effects than other studied fractions, such as the hexane (Hex) and aqueous (Aq) fractions [20]. The yield of DCM and EtOAc fractions was 6.2 g and 3.2 g, respectively. Additional subfractions were obtained (subfractions (A-M) from the DCM fraction and subfractions (A-H) from the EtOAc fraction) [20]. The isolated compounds were obtained from the F subfraction (661.0 mg) of the DCM fraction and from the D subfraction (2.5 g) of the EtOAc fraction. The DCM fraction yielded 76.3 mg of compound 1 (noreugenin); the EtOAc fraction yielded 61.9 mg of a 1 : 1 mixture of the compounds 2 (orobol) and 3 (butein) and 122.7 mg of compound 4 (*α*-hydroxy-butein). The chemical identity of the isolated compounds was established by analyzing their spectral data (^1^H NMR, correlation spectroscopy (COSY), Heteronuclear Single Quantum Correlation (HSQC), and Heteronuclear Multiple Bond Correlation (HMBC)), as previously reported by Lima and collaborators [16].

### 2.3. Animals

For the experiments, male and female Swiss mice (18–22 g, 1 month old) were used. The animals received free access to standard mouse chow (Nuvilab CR-1™, Nuvital, São Paulo, Brazil) and water *ad libitum*. They were housed under standardized conditions in a room at a constant temperature (20 ± 2°C), with alternating 12 h periods of light and darkness and 50–60% humidity. In this study, a minimum number of animals were used to have a consistent statistical analysis. This protocol was approved by the Committee for Ethics in Animal Research of the Federal University of Santa Catarina (CEUA-Protocol PP00757/CEUA/2012), and the experiments were performed in accordance with the norms of the National Council of Animal Experimentation Control (CONCEA).

### 2.4. *Ex Vivo* Assays

#### 2.4.1. Neutrophil Isolation

Neutrophils were obtained from male and female Swiss mice 4 h after an intraperitoneal (i.p.) injection of 3 mL of sterile oyster glycogen solution (1%) dissolved in sterile phosphate-buffered saline (PBS, pH 7.6, composition: 130 mmol NaCl, 5 mmol Na_2_HPO_4_, and 1 mmol KH_2_PO_4_). After this period, the animals were anesthetized and euthanized with an overdose of pentobarbital (120 mg/kg) administered subcutaneously (s.c.). The cells were collected by rinsing the abdominal cavity with 3 mL of sterile PBS, pH 7.6 [21].

#### 2.4.2. Neutrophil Viability

Neutrophils were obtained from the fluid leakage of the mouse peritoneal lavage, washed with PBS (pH 7.6), and the mixture was centrifuged at 900 × *g* for 10 min at 4°C (Sorvall™ ST 40, Thermo Scientific®, Swedesboro, NJ, USA). After washing, the neutrophils were suspended in PBS (pH 7.6) and 30 *μ*L of this cell suspension (1 × 10^6^) was incubated with 10 *μ*L of different doses of noreugenin (NRG: 1, 2.5, 5, 10, 50, or 100 *μ*M) or *α*-hydroxy-butein (AH-BU: 1, 2.5, 5, 10, 50, or 100 *μ*M) in the presence or absence of lipopolysaccharide (LPS: 5 *μ*g/mL, 10 *μ*L) (LPS from *Escherichia coli* serotype O111:B4, Sigma-Aldrich, St. Louis, MO, USA), for 18 h in an enzyme immunoassay (EIE) plate [21–23]. For the next step, 10 *μ*L of neutrophil suspension from each sample of the different doses of the compounds was mixed with 10 *μ*L of trypan blue dye solution (0.4%). The suspension (10 *μ*L) was placed in a Neubauer chamber, 100 cells were counted, and the percentage of viable cells was calculated.

#### 2.4.3. Determination of Myeloperoxidase Activity

Neutrophils obtained from the fluid leakage of the mouse peritoneal lavage were incubated with different doses of noreugenin (NRG: 1, 2.5, 5, 10, or 50 *μ*M) or *α*-hydroxy-butein (AH-BU: 1, 2.5, 5, 10, or 50 *μ*M) in the absence or presence of LPS (5 *μ*g/mL) for 18 h in an EIE plate. The mixture was centrifuged at 900 × *g* for 5 min at 4°C (Sorvall™ ST 40, Thermo Scientific®, Swedesboro, NJ, USA). The neutrophil culture supernatant was used to measure MPO *activity* by a colorimetric assay, using the following procedure: the in-house assay method described in the literature was used to determine the MPO *activity* [24]. For this determination, 20 *μ*L of each sample supernatant was added to 180 *μ*L of buffer solution (composition: 0.167 mg/mL o-dianisidine dihydrochloride and 0.0005% H_2_O_2_), transferred to the EIE plate, and incubated at 37°C for 15 min. After this time, 15 *μ*L of a stop solution (sodium azide, 1%) was added to the each well in the EIE plate. The colorimetric assay was performed using an EIE plate reader (Organon Teknika, Roseland, NJ, USA) at 450 nm and interpolated from a standard MPO curve (0.7–140 mU/mL, using myeloperoxidase from human leukocytes (Sigma-Aldrich, St. Louis, MO, USA)). The results were expressed in mU of MPO/mL, whereby 1 unit of MPO was defined as the amount of enzyme degrading 1 nmol H_2_O_2_ per min at 37°C.

#### 2.4.4. Determination of Neutrophil Apoptosis and Necrosis

To analyze the apoptosis and necrosis of neutrophils, the cells were obtained from the fluid leakage of the mouse peritoneal lavage and were incubated with one dose of noreugenin (5 *μ*M) or *α*-hydroxy-butein (5 *μ*M) in the absence or presence of LPS (5 *μ*g/mL) for 18 h in the EIE plate.

Subsequently, cells were washed with cold PBS (pH 7.6) and centrifuged at 900 × *g* for 5 min at 4°C (Sorvall™ ST 40, Thermo Scientific®, Swedesboro, NJ, USA). The supernatant was discarded, and the pellet was washed twice with PBS, pH 7.6 (300 *μ*L). The subsequent pellet was suspended in 300 *μ*L of binding buffer (composition: 10 mM hydroxyethyl piperazine ethanesulfonic acid, pH 7.4, composition: 150 mM NaCl, 5 mM KCl, 1 mM MgCl_2_, and 1.8 mM CaCl_2_), and the cells were stained with the following antibodies: (1) 2.0 *μ*L of Ly6G conjugated with phycoerythrin (PE) (BD Biosciences, San Jose, CA, USA); (2) 2.0 *μ*L of CD11b conjugated with PE-Cy™7 (BD Biosciences, San Jose, CA, USA); (3) 2.0 *μ*L of F4/80 conjugated with PerCP (BD Biosciences, San Jose, CA, USA); and (4) 2.5 *μ*L of Annexin V conjugated with fluorescein isothiocyanate (FITC) (BD Biosciences, San Jose, CA, USA), and with 2.5 *μ*L of 7-AAD (BD Biosciences, San Jose, CA, USA) to analyze apoptosis and necrosis of the neutrophils, according to the manufacturer's instructions. Cells were immediately analyzed by a FACSVerse® flow cytometer (BD Biosciences, San Jose, CA, USA) using the FACSuite® software, and data from 10,000 events were obtained. The results for each population were expressed as percentages.

### 2.5. *In Vivo* Assay

#### 2.5.1. Carrageenan-Induced Pleurisy

Pleurisy was induced by a single intrapleural (i.pl.) injection of 0.1 mL of Cg (1%) according to previously described methods [25]. Four hours after the induction of pleurisy, the mice were euthanized with an overdose of pentobarbital (120 mg/kg, i.p.). The thorax was opened, and the pleural cavity was exposed and washed with 1.0 mL of PBS (pH 7.6) with heparin (20 UI/mL). The fluid leakage from the pleural cavity of different groups of mice was used to quantify cytokine (IL-1*β*, IL-17A, and IL-10) levels and lipid peroxidation (TBARS concentration) as well as superoxide dismutase (SOD), catalase (CAT), and glutathione S-transferase (GST) activities.

#### 2.5.2. Experimental Design

Based on the data published by Rosa et al. [20], we selected single doses for the NRG (5 mg/kg) or AH-BU (2.5 mg/kg) administrations 0.5 h before carrageenan-induced pleurisy to analyze their effects on IL-1*β*, IL-17A, and IL-10 levels as well as on TBARS concentration and antioxidant enzyme activities (SOD, CAT, and GST) in the fluid leakage from the mouse pleural cavity. These doses were chosen because they presented better anti-inflammatory actions by inhibiting leukocytes and exudate concentrations in a mouse model of carrageenan-induced pleurisy [20].

Dexamethasone (Dex: 0.5 mg/kg), administered i.p. 0.5 h before the induction of pleurisy, was used as a reference anti-inflammatory drug. In addition, a positive control group (animals treated with Cg i.pl. only) and a negative control group (animals treated with sterile saline i.pl. (NaCl, 0.9%) only) were included in all groups of *in vivo* experiments.

#### 2.5.3. Quantification of IL-1*β*, IL-17A, and IL-10 Levels

The levels of inflammatory cytokines (IL-1*β*, IL-17A, and IL-10) in the fluid leakage from the mouse pleural cavity were determined using commercial kits containing monoclonal specific antibodies for each cytokine. The cytokine levels were measured using an EIE kit according to the manufacturer's instructions (IL-1*β*, eBioscience, Inc., San Diego, CA, USA; IL-17A, eBioscience, Inc., San Diego, CA, USA; IL-10, eBioscience, Inc., San Diego CA, USA). All cytokine levels were estimated by means of colorimetric measurements at 450 nm and by interpolation from a standard curve of an EIE plate reader (Organon Teknika, Roseland, NJ, USA). The results are expressed in pg/mL.

#### 2.5.4. Quantification of Lipid Peroxidation

Lipid peroxidation was evaluated in the fluid leakage from the mouse pleural cavity by determination of concentration of thiobarbituric acid reactive substances (TBARS) [26]. In this protocol, 100 *μ*L of mouse pleural fluid leakage was precipitated with 1 mL of trichloroacetic acid (TCA) (12%) followed by incubation with 1 mL of thiobarbituric acid (TBA) (0.73%), in 0.9 mL of buffer containing 60 mM Tris–HCl and 0.1 mM DPTA, pH 7.4, at 100°C, for 60 min. After the samples were then centrifuged (5 min, 1500 *g*), the absorbance of the pink chromophore was measured in the supernatant spectrophotometrically at 535 nm with an EIE plate reader (SpectraMax Paradigm, Molecular Devices®, Sunnyvale, CA, USA). The concentrations of TBARS were expressed in nmol TBARS/mL.

#### 2.5.5. Catalase Activity

The CAT activity was determined in the fluid leakage from the mouse pleural cavity according to the method described by Aebi [27]. The reaction is based on the measurement of the decreased absorbance of hydrogen peroxide (H_2_O_2_) solution freshly prepared (dissolved in PBS, pH 7.0, composition: 10 mM H_2_O_2_, 130 mmol NaCl, 5 mmol Na_2_HPO_4_, and 1 mmol KH_2_PO_4_) in the presence of the enzyme CAT in the mouse pleural fluid leakage. Briefly, 5 *μ*L of mouse pleural fluid leakage was used and the reaction was initiated by adding 200 *μ*L of H_2_O_2_ solution (dissolved in PBS, pH 7.0) to the EIE plate. The CAT activity was measured as the change in optical density every 30 s for 3 min at 240 nm with the EIE plate reader (SpectraMax Paradigm, Molecular Devices®, Sunnyvale, CA, USA). The enzyme activity was expressed in mmol H_2_O_2_/min/mL.

#### 2.5.6. Superoxide Dismutase Activity

Superoxide dismutase (SOD) activity was analyzed in the fluid leakage from the mouse pleural cavity in accordance to the method described by Misra and Fridovich and modified by Boveris et al. [28, 29]. The reaction is based on epinephrine oxidation (pH 2.0 to pH 10.2), which produces superoxide anion radicals and adrenochrome. In this protocol, the epinephrine–adrenochrome transition was inhibited by the superoxide dismutase, which was present in the mouse pleural fluid leakage. Briefly, 5 *μ*L, 10 *μ*L, 20 *μ*L, and 40 *μ*L of the pleural fluid leakage were added to 200 *μ*L of 50 mM glycine solution (dissolved in H_2_O, pH 10.2). The reaction was started by the addition of 5 *μ*L of freshly prepared 60 mM epinephrine (pH ~2.0) to the mixture in the EIE plate. The absorbance was observed every 20 s during 4 min at 480 nm by the EIE plate reader (SpectraMax Paradigm, Molecular Devices®, Sunnyvale, CA, USA). The unit of enzyme activity was expressed as 50% of auto-oxidation inhibition of epinephrine–adrenochrome, and the result was expressed in USOD/mL.

#### 2.5.7. Glutathione S-Transferase Activity

Glutathione S-transferase (GST) activity was measured in the fluid leakage from the mouse pleural cavity in accordance to the methodology described by Habig et al. [30]. In this reaction, 1-chloro-2,4-dinitrobenzene (CDNB; as substrate for GST) was used. In this protocol, GST with reduced glutathione (GSH) promotes the CDNB–GSH conjugation. Briefly, 5 *μ*L of sample was added to the 260 *μ*L reaction mixture containing 250 *μ*L PBS (pH 7.0), 5 *μ*L CDNB, and 5 *μ*L GSH solution in the EIE plate. The absorbance was monitored every 20 s during 1 min at 340 nm by the EIE plate reader (SpectraMax Paradigm, Molecular Devices®, Sunnyvale, CA, USA). The enzyme activity was expressed in *μ*mol/min/mL.

### 2.6. Statistical Analysis

The results of the *in vivo* experiments are presented as mean ± standard error of the mean (S.E.M.) and of the *ex vivo* experiments as mean ± S.E.M or percentage (%). All the data were analyzed statistically by analysis of variance (ANOVA) complemented with the Newman–Keuls post hoc test and/or Student's *t*-test when necessary. Values of *p* ≤ 0.05 were considered significant.

### 2.7. Drugs and Reagents

The following drugs and reagents used carrageenan (degree IV), human neutrophil myeloperoxidase, o-dianisidine dihydrochloride, trypan blue dye, oyster glycogen, and lipopolysaccharide were purchased from Sigma Chemical Co. (St Louis, MO, USA). Dexamethasone was purchased from Ache Pharmaceutical Laboratories S.A., São Paulo, SP, Brazil. The chemical reagents were purchased from Vetec, Rio de Janeiro, RJ, Brazil, and Reagen, Rio de Janeiro, RJ, Brazil. The enzyme-linked immunosorbent assay (ELISA: EIA) for the quantification of mouse interleukin 1*β*, interleukin 17A, and interleukin 10 was purchased from eBioscience, Inc., San Diego, CA, USA. The antibodies (Ly6G conjugated with phycoerythrin (PE), CD11b conjugated with PE-Cy™7, Annexin V conjugated with fluorescein isothiocyanate (FITC), and 7-AAD) used in the apoptosis/necrosis assay were purchased from BD Biosciences, San Jose, CA, USA. All other reagents used were of analytical grade and obtained from various commercial sources.

## 3. Results

### 3.1. Phytochemical Analysis of Isolation and Identification of the Compounds

**Figure 1:**
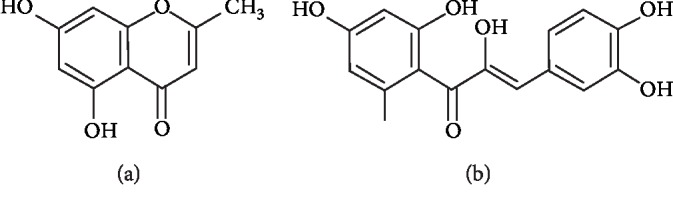
Chemical structure of isolated compounds: noreugenin (a) and *α*-hydroxy-butein (b) from *C. uniflora* Less.

The chromatographic separation of the DCM and EtOAc fractions of *Calea uniflora* Less. led to the isolation, characterization, and identification of the four major compounds: (1) noreugenin, a mixture of (2) orobol + (3) butein, and (4) *α*-hydroxy-butein (Figure 1); two of them (noreugenin and *α*-hydroxy-butein) were isolated with high grade of purity (>99%) and submitted to the *ex vivo* and *in vivo* experiments. The structures of the isolated compounds were established *via* analysis of their spectral data (^1^H NMR, COSY, HSQC, and HMBC) as previously published by Lima et al. [16].

### 3.2. *Ex Vivo* Assays

#### 3.2.1. Effect of Noreugenin and *α*-Hydroxy-Butein on Neutrophil Viability

**Figure 2:**
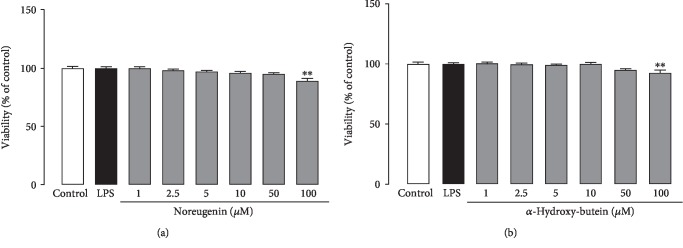
Effect of noreugenin (a) and *α*-hydroxy-butein (b) on cell viability of murine neutrophils. Vehicle = the isolated neutrophils treated only with sterile phosphate-buffered saline (PBS, pH 7.6). LPS = cells treated with LPS (5 *μ*g/mL) only. Noreugenin = cells treated with noreugenin (1-100 *μ*M)+LPS (5 *μ*g/mL). *α*-Hydroxy-butein = cells treated with *α*-hydroxy-butein (1-100 *μ*M)+LPS (5 *μ*g/mL). Each group represents the mean ± S.E.M. of experiments conducted in triplicate from three to six animals. ^∗∗^*p* < 0.01 compared to the control group (Control); ANOVA/Newman–Keuls test.

To study the cytotoxicity of the isolated compounds, the neutrophils were incubated with different concentrations of NRG (1 *μ*M to 100 *μ*M) or AH-BU (1 *μ*M to 100 *μ*M) with LPS (5 *μ*g/mL) for 18 h. The results showed that NRG and AH-BU at concentrations up to 50 *μ*M did not affect the cell viability (*p* > 0.05) (Figure 2). Cells treated with 100 *μ*M of NRG or AH-BU showed reduced cell viability (*p* < 0.01) (Figure 2).

#### 3.2.2. Effect of Noreugenin and *α*-Hydroxy-Butein on Myeloperoxidase Activity

For MPO quantification, the tested concentrations of the isolated compounds were 1-50 *μ*M, because the concentration of 100 *μ*M reduced neutrophil viability.

In this study, the NRG (5–50 *μ*M) and AH-BU (5 *μ*M–50 *μ*M) significantly decreased MPO *activity* in the primary culture of neutrophils (*p* < 0.01) (Table 1).

#### 3.2.3. Effect of Noreugenin and *α*-Hydroxy-Butein on Neutrophil Apoptosis and Necrosis

**Figure 3:**
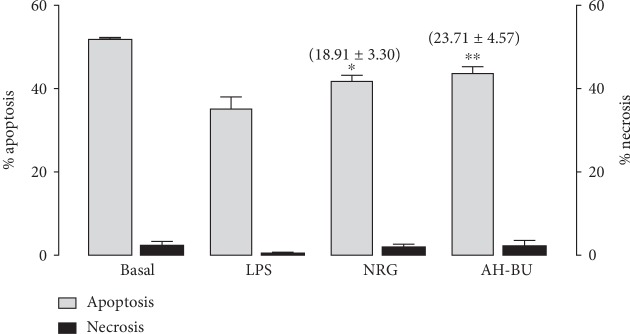
Effect of noreugenin or *α*-hydroxy-butein on neutrophilic apoptosis in carrageenan-induced inflammation in the mouse model of pleurisy. Neutrophils were characterized in flow cytometry by expression of Ly6G and CD11b and absence of F4/80 in their surface. Basal = cells treated with sterile phosphate-buffered saline (PBS, pH 7.6) only. LPS = cells treated with LPS (5 *μ*g/mL) only. NRG = cells treated with noreugenin (5 *μ*M)+LPS (5 *μ*g/mL). AH-BU = cells treated with *α*-hydroxy-butein (5 *μ*M)+LPS (5 *μ*g/mL). *Bars* represent the mean ± S.E.M. of experiments conducted in triplicate from three to six animals. The values in brackets represent the percentages of inhibition. ^∗^*p* < 0.05 and ^∗∗^*p* < 0.01 compared to the positive control group (LPS); ANOVA/Newman–Keuls test.

To evaluate the type of cell death of neutrophils (apoptosis or necrosis), the lowest dose of the NRG and AH-BU that caused the most significant inhibition of the MPO *activity* was selected. The results demonstrated that NRG (5 *μ*M) and AH-BU (5 *μ*M) caused a significant increase in apoptosis of murine neutrophils (*p* < 0.05) (Figure 3).

### 3.3. *In Vivo* Assay

Considering the results found in the *in vitro* assays, we investigated the effect of NRG and AH-BU in an animal model of neutrophil inflammation (carrageenan-induced pleurisy). For this, we analyzed whether the anti-inflammatory effect of the isolated compounds could be related to the modulation of other important mediators involved in the inflammatory response, such as proinflammatory cytokines and oxidative stress.

#### 3.3.1. Effect of Noreugenin and *α*-Hydroxy-Butein on IL-1*β*, IL-17A, and IL-10 Levels

**Figure 4:**
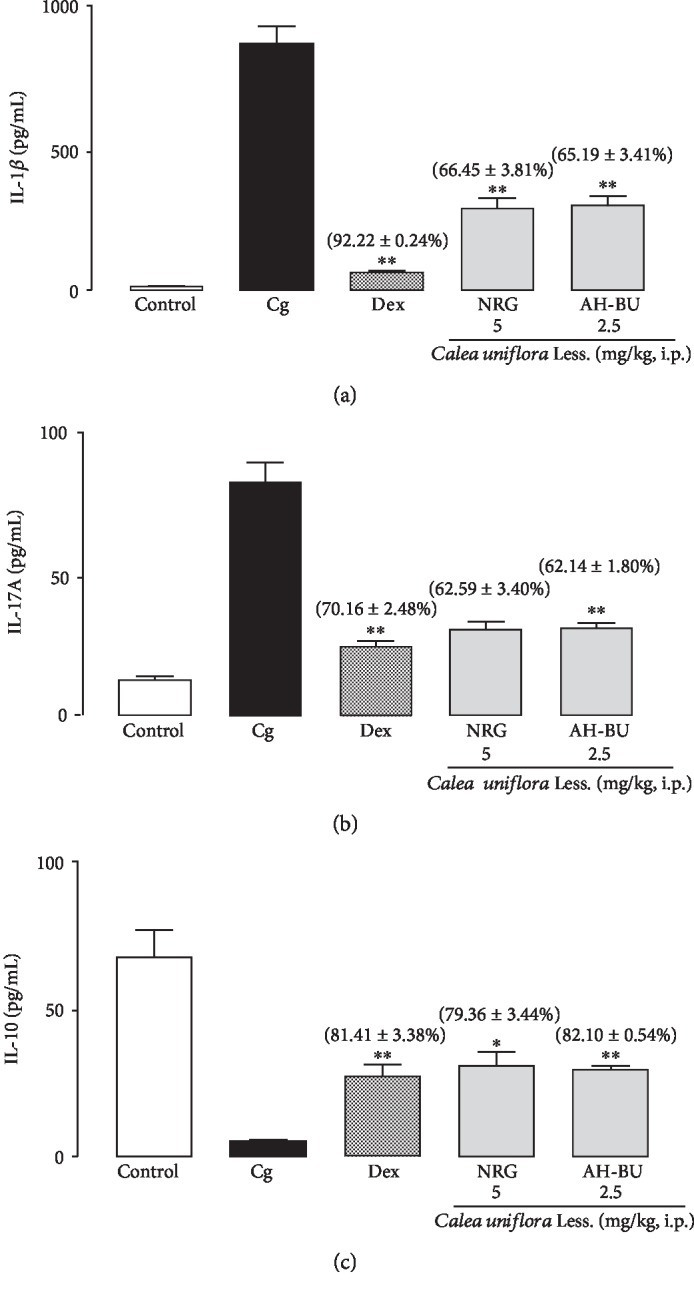
Effect of noreugenin or *α*-hydroxy-butein upon IL-1*β* (a), IL-17A (b), and IL-10 (c) levels in carrageenan-induced inflammation in the mouse model of pleurisy. Control = animals treated with saline solution (NaCl 0.9%) only. Cg = animals treated with carrageenan (1%) only. Dex = animals pretreated with dexamethasone (0.5 mg/kg, i.p.). NRG = noreugenin (5 mg/kg, i.p.). AH-BU = *α*-hydroxy-butein (2.5 mg/kg, i.p.). *Bars* represent the mean ± S.E.M. of 5 animals. The values in brackets represent the percentages of inhibition. ^∗^*p* < 0.05 and ^∗∗^*p* < 0.01 compared to the positive control group (Cg); ANOVA/Newman–Keuls test.

The results demonstrated that NRG (5 mg/kg) and AH-BU (2.5 mg/kg) caused a significant decrease in the levels of certain cytokines (order of inhibition: IL-1*β* > IL-17A) (*p* < 0.01). On the other hand, it was observed that the isolated compounds caused a significant increase in IL-10 levels (*p* < 0.05) (Figure 4). Dex also significantly inhibited IL-1*β* and IL-17A levels and increased IL-10 levels (*p* < 0.01) (Figure 4).

#### 3.3.2. Effect of Noreugenin and *α*-Hydroxy-Butein on Lipid Peroxidation

NRG (5 mg/kg) and AH-BU (2.5 mg/kg) caused a significant reduction in concentration of TBARS at 4 h of the inflammatory process induced by carrageenan (*p* < 0.05) (Table 2). Similarly, Dex also inhibited this inflammatory parameter (*p* < 0.01) (Table 2).

#### 3.3.3. Effect of Noreugenin and *α*-Hydroxy-Butein on Antioxidant Enzyme (CAT, SOD, and GST) Activities

The results showed that activities of antioxidant enzymes (CAT, SOD, and GST) were markedly reduced by NRG (5 mg/kg) and AH-BU (2.5 mg/kg) (*p* < 0.05) (Table 2). In addition, Dex suppressed these antioxidant enzyme activities (*p* < 0.05) (Table 2).

## 4. Discussion

Neutrophils play a relevant role in the defense against pathogens. They are rapidly recruited to the site of the inflammatory response or lesion; they phagocytose and kill pathogens using different mechanisms, including the release of enzymes, cytokines, neutrophil extracellular traps, and reactive oxygen species [31, 32].

However, the neutrophils can also persist beyond the acute phase of inflammation and, in this case, they are actively involved in chronic inflammation and may lead to host tissue damage [33]. Hence, delayed neutrophil death can cause tissue damage by the generation of reactive oxygen species (ROS). Thus, delayed neutrophil death can be modulated by the induction of apoptosis or necrosis. The ideal scenario for the resolution of inflammation is to stop neutrophil activation by the induction of neutrophil apoptosis after they phagocytose dead bacteria [34, 35]. Thus, the control of neutrophils at the site of injury may prove to be a potential therapeutic target.

In the present study, the compounds NRG and AH-BU extracted from *Calea uniflora* Less. were evaluated for their effect on neutrophils activated by LPS, which induces the production/release of a variety of inflammatory mediators, such as MPO.

MPO is an important enzyme associated with leukocyte recruitment, and it reflects neutrophil activity [36]. The results obtained in the present study show that the phenolic compounds NRG and AH-BU were effective in reducing MPO *activity* in LPS-activated neutrophils. This is a strong indication that these isolated compounds reduced the neutrophil activation at the site of inflammation. These results obtained corroborate with data from the literature, such as the previous study developed in our research group, which demonstrated that NRG and AH-BU were potent compounds for the reduction of MPO *activity* in a mouse model of carrageenan-induced pleurisy [20]. This decrease in MPO *activity* caused by isolated compounds led us to investigate whether this effect could be related to neutrophil apoptosis. On the contrary, the results showed that NRG and AH-BU increased neutrophil apoptosis compared to cells treated with LPS only. In addition, it is important to emphasize that NRG and AH-BU had noncytotoxic effects on neutrophils.

Based on the results obtained from the *ex vivo* assays, we proceeded to evaluate these compounds *in vivo*. Here, we evaluated the effects of NRG and AH-BU on pro- and anti-inflammatory cytokines and oxidative stress in the inflamed pleural exudates induced by carrageenan in mice. This is a useful tool to screen for new anti-inflammatory drugs, particularly those derived from medicinal plants [25].

Proinflammatory cytokines, such as IL-17A and IL-1*β*, are released during neutrophil activation [37]. In this study, these cytokine levels were reduced by NRG and AH-BU, which are consistent with the results of another study conducted by Nader et al. [38]. This research group demonstrated that *Jungia sellowii*, a species also belonging to the Asteraceae family, reduced IL-1*β* and IL-17A levels as well as neutrophil infiltration in a murine model of carrageenan-induced lung inflammation [38].

Moreover, our results are also supported by Menegazzi et al. [39] and Ahmad et al. [40], who demonstrated that phenolic compounds reduced the infiltration of neutrophils, production of tumor necrosis factor alpha (TNF-*α*), IL-1*β*, and IL-17A, as well as the release of other proinflammatory cytokines in a mouse model of carrageenan-induced pleurisy [39, 40].

In parallel, under the same experimental conditions, NRG and AH-BU also promoted the increase in anti-inflammatory cytokine (IL-10) levels in the fluid leakage of the mouse pleural cavity. Similarly, a study conducted by Ahmad et al. [40] demonstrated that phenolic compounds were effective in inhibiting the production of proinflammatory mediators such as interleukin 6 (IL-6), interferon gamma (IFN-*γ*), IL-17A, and TNF-*α* and stimulating the secretion of anti-inflammatory mediators such as IL-10, interleukin 13 (IL-13), and transforming growth factor beta-1 (TGF-*β*1) in pleural exudates in a mouse model of carrageenan-induced pleurisy [40].

Oxidative stress refers to the excessive production of ROS in the cells and tissues, which may cause tissue injury and lead to the inflammatory process. In comparison to other phagocytes, neutrophils generate high concentrations of ROS. Among the group of exogenous antioxidants (i.e., dietary), the phenolic compounds present in plants have the ability to suppress ROS formation by either inhibition of enzymes involved in their production, scavenging of ROS, upregulation, or protection of antioxidant defenses [19, 41, 42].

Corroborating with the literature, the present study showed that the phenolic compounds NRG and AH-BU were effective in reducing the activities of important enzymes such as SOD, CAT, and GST. These enzymes participate in the main defense system against oxidative stress induced by an increased generation of ROS, consequently inhibiting the inflammatory process [41, 42]. Therefore, we might assume that the phenolic compounds NRG and AH-BU, which were utilized in the current study, acted as antioxidants to suppress the production of oxygen radicals, resulting in a decrease in antioxidant enzyme activities. However, we observed that these compounds were effective in reestablishing the activities of CAT, SOD, and GST at the baseline so that the activities of these enzymes were similar to those of animals treated with saline only (negative control group).

A primary consequence of oxidative stress and increased levels of reactive species caused by inflammatory process is the oxidation of proteins and lipids, the latter also known as lipid peroxidation [43].

In the present study, treatment with NRG and AH-BU decreased the lipid peroxidation, as evaluated indirectly by the reduction in TBARS concentration. These results are also in accordance with those from other reports, which demonstrated that activated neutrophils migrate to the inflamed paw, release enzymes (e.g., MPO), and increase ROS production in paw edema induced by the carrageenan test [44]. Furthermore, the treatment of animals with polyphenolic phytochemicals modulates inflammation by reducing TBARS, MPO *activity*, and infiltration of neutrophils [44].

## 5. Conclusions

In summary, the results of this study demonstrated that NRG and AH-BU exhibit important anti-inflammatory and antioxidant properties. The isolated compounds showed a significant anti-inflammatory effect by inhibiting either a proinflammatory enzyme (MPO) or cytokines (IL-1*β* and IL-17A) and increasing both an anti-inflammatory cytokine (IL-10) and neutrophil apoptosis. These effects were probably associated with the reduction of the reactive species generation, observed indirectly by reestablishment of antioxidant enzyme activities. These compounds could prove to be potential novel lead compounds for the development of anti-inflammatory drugs in the future.

## Figures and Tables

**Table 1:** Effects of noreugenin (NRG) or *α*-hydroxy-butein (AH-BU) on myeloperoxidase activity of the neutrophils obtained from fluid leakage from the mouse peritoneal cavity 4 h after an intraperitoneal injection of oyster glycogen solution.

Groups	Concentrations (*μ*m)	MPO (mU/mL) (% of inhibition)
Control		371.10 ± 3.30
LPS		437.80 ± 5.80

Noreugenin	1	423.10 ± 4.21
	2.5	417.30 ± 7.53
	5	391.90 ± 10.76(10.47±2.46%)^∗∗^
	10	373.90 ± 11.48(14.60±2.62%)^∗∗^
	50	369.20 ± 5.32(15.68±1.21%)^∗∗^

*α*-Hydroxy-butein	1	418.40 ± 6.92
	2.5	418.40 ± 6.88
	5	379.90 ± 7.82(13.23±1.79%)^∗∗^
	10	371.00 ± 7.50(15.26±1.71%)^∗∗^
	50	365.80 ± 3.15(16.46±0.72%)^∗∗^

MPO: myeloperoxidase; LPS: lipopolysaccharide. Cells were pretreated with different concentrations of noreugenin (NRG: 1–50 *μ*M) or *α*-hydroxy-butein (AH-BU: 1–50 *μ*M) for 0.5 h and then stimulated with LPS (5 *μ*g/mL) for 18 h. Control = cells treated with sterile phosphate-buffered saline (PBS, pH 7.6) only. LPS = cells treated with LPS (5 *μ*g/mL) only. Each group represents the mean ± S.E.M. of experiments conducted in triplicate from three to six animals. ^∗∗^*p* < 0.01 compared to the positive control group (LPS); ANOVA/Newman–Keuls test.

**Table 2:** Effects of noreugenin (NRG) and *α*-hydroxy-butein (AH-BU) on antioxidant enzyme activities and TBARS concentration in carrageenan-induced inflammation in the mouse model of pleurisy.

Groups/dose (mg/kg)	CAT (mmol/min/mL) (% of inhibition)	SOD (USOD/mL) (% of inhibition)	GST (*μ*mol/min/mL) (% of inhibition)	TBARS (mmol/mL) (% of inhibition)
Sal^a^	10.91 ± 1.20	31.45 ± 3.73	6.90 ± 1.10	2.81 ± 0.21
Cg^a^	31.61 ± 4.22	63.75 ± 3.19	16.36 ± 0.59	10.64 ± 0.58
Dex (0.5)^b^	14.05 ± 0.63(55.54±1.99)^∗∗^	40.90 ± 3.63(35.84 ± 5.70)^∗^	9.57 ± 0.74(41.49±4.52)^∗∗^	4.13 ± 0.85(61.18±7.96)^∗∗^
NRG (5)^b^	12.04 ± 1.23(61.93±3.88)^∗∗^	39.55 ± 4.09(37.96 ± 6.42)^∗^	4.23 ± 0.29(74.15±1.80)^∗∗^	5.88 ± 0.96(44.72±9.02)^∗∗^
AH-BU (2.5)^b^	13.23 ± 2.13(58.16±6.74)^∗∗^	26.43 ± 5.68(58.55±8.91)^∗∗^	5.31 ± 0.94(67.56±5.74)^∗∗^	6.00 ± 0.57(43.61 ± 5.36)^∗^

CAT: catalase; SOD: superoxide dismutase; GST: glutathione S-transferase; TBARS: thiobarbituric acid reactive substances. Noreugenin (NRG: 5 mg/kg) and *α*-hydroxy-butein (AH-BU: 2.5 mg/kg) administered 0.5 h before pleurisy induction by carrageenan (1%). Sal = animals treated with sterile saline solution (NaCl 0.9%) only. Cg = animals treated with carrageenan (1%) only. Dex = animals pretreated with dexamethasone (0.5 mg/kg). ^a^Administered by intrapleural injection (i.pl.). ^b^Administered by intraperitoneal route (i.p.). Each group represents the mean ± S.E.M. of 5 animals. ^∗^*p* < 0.05 and ^∗∗^*p* < 0.01 compared to the positive control group (Cg); ANOVA/Newman–Keuls test.

## Data Availability

No data were used to support this study.

## Abbreviations

AH-BU: *α*-Hydroxy-butein
Aq: Aqueous
*C. uniflora*: *Calea uniflora* Less.
CAT: Catalase
CC: Column chromatography
CDNB: 1-Chloro-2,4-dinitrobenzene
Cg: Carrageenan
CONCEA: National Council of Animal Experimentation Control
COSY: Correlation spectroscopy
DCM: Dichloromethane
Dex: Dexamethasone
EIE: Enzyme-linked immunosorbent assay
EtOAc: Ethyl acetate
FITC: Fluorescein isothiocyanate
GSH: Glutathione
GST: Glutathione S-transferase
Hex: Hexane
HMBC: Heteronuclear Multiple Bond Correlation
HSQC: Heteronuclear Single Quantum Correlation
IFN-*γ*: Interferon gamma
IL-1*β*: Interleukin 1 beta
IL-6: Interleukin 6
IL-10: Interleukin 10
IL-13: Interleukin 13
IL-17A: Interleukin 17A
i.p.: Intraperitoneal
i.pl.: Intrapleural
LPS: Lipopolysaccharide
MPO: Myeloperoxidase
NMR: Nuclear magnetic resonance
NRG: Noreugenin
OR+BU: Orobol+butein
ROS: Reactive oxygen species
s.c.: Subcutaneous route
SOD: Superoxide dismutase
TBA: Thiobarbituric acid
TBARS: Thiobarbituric acid reactive substances
TCA: Trichloroacetic acid
TGF-*β*1: Transforming growth factor beta-1
TLC: Thin-layer chromatography
TNF-*α*: Tumor necrosis factor alpha
VLC: Vacuum liquid chromatography

## References

[1] Chovatiya R., Medzhitov R. (2014). Stress, inflammation, and defense of homeostasis. *Molecular Cell*.

[2] Ashley N. T., Weil Z. M., Nelson R. J. (2012). Inflammation: mechanisms, costs, and natural variation. *Annual Review of Ecology, Evolution, and Systematics*.

[3] Headland S. E., Norling L. V. (2015). The resolution of inflammation: principles and challenges. *Seminars in Immunology*.

[4] Biswas S. K. (2016). Does the interdependence between oxidative stress and inflammation explain the antioxidant paradox?. *Oxidative Medicine and Cellular Longevity*.

[5] Dalmarco E. M., Budni P., Parisotto E. B., Wilhelm-Filho D., Fröde T. S. (2009). Antioxidant effects of mycophenolate mofetil in a murine pleurisy model. *Transplant Immunology*.

[6] Hussain T., Tan B., Yin Y., Blachier F., Tossou M. C. B., Rahu N. (2016). Oxidative stress and inflammation: what polyphenols can do for us?. *Oxidative Medicine and Cellular Longevity*.

[7] Kobayashi S. D., Malachowa N., Deleo F. R. (2017). Influence of microbes on neutrophil life and death. *Frontiers in Cellular and Infection Microbiology*.

[8] Kolaczkowska E., Kubes P. (2013). Neutrophil recruitment and function in health and inflammation. *Nature Reviews Immunology*.

[9] Teng T., Ji A., Ji X., Li Y. (2017). Neutrophils and immunity: from bactericidal action to being conquered. *Journal of Immunology Research*.

[10] Antman E. M., Bennett J. S., Daugherty A., Furberg C., Roberts H., Taubert K. A. (2007). Use of nonsteroidal antiinflammatory drugs: an update for clinicians: a scientific statement from the American Heart Association. *Circulation*.

[11] Rhen T., Cidlowski J. A. (2005). Antiinflammatory action of glucocorticoids: new mechanisms for old drugs. *The New England Journal of Medicine*.

[12] Alessandri A. L., Sousa L. P., Lucas C. D., Rossi A. G., Pinho V., Teixeira M. M. (2013). Resolution of inflammation: mechanisms and opportunity for drug development. *Pharmacology & Therapeutics*.

[13] Gilroy D., Maeyer R. (2015). New insights into the resolution of inflammation. *Seminars in Immunology*.

[14] Ferraz A. B. F., Pinheiro S. P., Oliveira P. A., Lino F. L., Picada J. N., Pereira P. (2009). Pharmacological and genotoxic evaluation of Calea clematidea and Calea uniflora. *Latin American Journal of Pharmacy*.

[15] Zank S., Hanazaki N. (2012). Exploring the links between ethnobotany, local therapeutic practices, and protected areas in Santa Catarina coastline, Brazil. *Evidence-based Complementary and Alternative Medicine*.

[16] Lima T. C., Souza R. J., Santos A. D. (2015). Evaluation of leishmanicidal and trypanocidal activities of phenolic compounds from *Calea uniflora* Less. *Natural Product Research*.

[17] Do Nascimento A. M., De Oliveira D. C. R., Albuquerque S. (2002). Evaluation of trypanocidal activity from *Calea uniflora* (Heliantheae-Asteraceae) extracts. *Revista Brasileira de Farmacognosia*.

[18] Segura-Cobos D., Venegas-Flores H., Baiza-Gutman L. A., Vazquez-Cruz B. (2010). Antinociceptive and anti-inflammatory effects of the methanol extract of *Calea zacatechichi* leaves and its fractions. *Pharmacology*.

[19] Zhang H., Tsao R. (2016). Dietary polyphenols, oxidative stress and antioxidant and anti-inflammatory effects. *Current Opinion in Food Science*.

[20] Rosa J. S., De Mello S. V. G. V., Vicente G. (2017). Calea uniflora Less. attenuates the inflammatory response to carrageenan- induced pleurisy in mice. *International Immunopharmacology*.

[21] Yamashita T., Ishibashi Y., Nagaoka I. (1982). Studies of glycogen-induced inflammation of mice. Dynamics of inflammatory responses and influence of antiinflammatory drugs and protease inhibitors. *Inflammation*.

[22] Stefani H. A., Botteselle G. V., Zukerman-Schpector J. (2012). Synthesis, anti-inflammatory activity and molecular docking studies of 2,5-diarylfuran amino acid derivatives. *European Journal of Medicinal Chemistry*.

[23] Santin J. R., Machado I. D., Drewes C. C. (2018). Role of an indole-thiazolidiene PPAR pan ligand on actions elicited by G-protein coupled receptor activated neutrophils. *Biomedicine & Pharmacotherapy*.

[24] Rao T. S., Currie J. L., Shaffer A. F., Isakson P. C. (1993). Comparative evaluation of arachidonic acid (AA) and tetradecanoylphorbol acetate (TPA)-induced dermal inflammation. *Inflammation*.

[25] Saleh T. S., Calixto J. B., Medeiros Y. S. (1996). Anti‐inflammatory effects of theophylline, cromolyn and salbutamol in a murine model of pleurisy. *British Journal of Pharmacology*.

[26] Bird R. P., Draper A. H. (1984). Comparative studies on different methods of malondialdehyde determination. *Methods in Enzymology*.

[27] Aebi H. (1984). *Catalase in vitro*. *Methods in Enzymology*.

[28] Misra H. P., Fridovich I. (1972). The univalent reduction of oxygen by reduced flavins and quinones. *The Journal of Biological Chemistry*.

[29] Boveris A., Fraga C. G., Varsavsky A. I., Koch O. R. (1983). Increased chemiluminescence and superoxide production in the liver of chronically ethanol-treated rats. *Archives of Biochemistry and Biophysics*.

[30] Habig W. H., Pabst M. J., Jacoby W. B. (1976). Glutathione-Stransferases: the first enzymatic step in mercapturic acid formation. *The Journal of Biological Chemistry*.

[31] Li J. L., Ng L. G. (2012). Peeking into the secret life of neutrophils. *Immunologic Research*.

[32] Garley M., Jabłońska E. (2017). Heterogeneity among neutrophils. *Archivum Immunologiae et Therapiae Experimentalis*.

[33] Pietrosimone K. M. (2015). Contributions of neutrophils to the adaptive immune response in autoimmune disease. *World Journal of Translational Medicine*.

[34] Bordon J., Aliberti S., Fernandez-Botran R. (2013). Understanding the roles of cytokines and neutrophil activity and neutrophil apoptosis in the protective versus deleterious inflammatory response in pneumonia. *International Journal of Infectious Diseases*.

[35] Kebir D. E., Filep J. (2013). Targeting neutrophil apoptosis for enhancing the resolution of inflammation. *Cell*.

[36] Fröde T. S., Medeiros Y. S. (2001). Myeloperoxidase and adenosine-deaminase levels in the pleural fluid leakage induced by carrageenan in the mouse model of pleurisy. *Mediators of Inflammation*.

[37] Amulic B., Cazalet C., Hayes G. L., Metzler K. D., Zychlinsky A. (2012). Neutrophil function: from mechanisms to disease. *Annual Review of Immunology*.

[38] Nader M., Vicente G., Da Rosa J. S. (2014). Jungia sellowii suppresses the carrageenan-induced inflammatory response in the mouse model of pleurisy. *Inflammopharmacology*.

[39] Menegazzi M., Di Paola R., Mazzon E. (2006). _Hypericum perforatum_ attenuates the development of carrageenan-induced lung injury in mice. *Free Radical Biology & Medicine*.

[40] Ahmad S. F., Zoheir K. M. A., Abdel-Hamied H. A. (2014). Grape seed proanthocyanidin extract protects against carrageenan-induced lung inflammation in mice through reduction of pro-inflammatory markers and chemokine expressions. *Inflammation*.

[41] Kasote D. M., Katyare S. S., Hegde M. V., Bae H. (2015). Significance of antioxidant potential of plants and its relevance to therapeutic applications. *International journal of biological sciences*.

[42] Warnatsch A., Tsourouktsoglou T., Branzk N. (2017). Reactive oxygen species localization programs inflammation to clear microbes of different size. *Immunity*.

[43] Thomas S. R., Witting P. K., Drummond G. R. (2008). Redox control of endothelial function and dysfunction: molecular mechanisms and therapeutic opportunities. *Antioxidants & Redox Signaling*.

[44] Nardi G. M., Januario A. G. F., Freire C. G. (2016). Anti-inflammatory activity of berry fruits in mice model of inflammation is based on oxidative stress modulation. *Pharmacognosy Research*.

